# A systematic review on the rotational thrombelastometry (ROTEM®) values for the diagnosis of coagulopathy, prediction and guidance of blood transfusion and prediction of mortality in trauma patients

**DOI:** 10.1186/s13049-016-0308-2

**Published:** 2016-10-03

**Authors:** Precilla V. Veigas, Jeannie Callum, Sandro Rizoli, Bartolomeu Nascimento, Luis Teodoro da Luz

**Affiliations:** 1 Department of Surgery, St. Michael’s Hospital and Institute of Medical Science, University of Toronto, 30 Bond Street, 3074 Donnelly Wing, Toronto, M5W 1B8 ON Canada; 2 Department of Clinical Pathology, Sunnybrook Health Sciences Center and Institute of Medical Science, University of Toronto, 2075 Bayview Avenue Room B2.04, Toronto, M4N 3M5 ON Canada; 3 Departments of Surgery and Critical Care Medicine, St. Michael’s Hospital and University of Toronto, 30 Bond Street, 3074 Donnelly Wing, Toronto, M5W 1B8 ON Canada; 4 Department of Surgery, Sunnybrook Health Sciences Centre and University of Toronto, 2075 Bayview Avenue, Room H1.71, Toronto, M4N 3M5 ON Canada

**Keywords:** Acute trauma coagulopathy, Thromboelastometry, Transfusion, Threshold, Bleeding

## Abstract

**Introduction:**

Viscoelastic assays have been promoted as an improvement over traditional coagulation tests in the management of trauma patients. Rotational thromboelastometry (ROTEM®) has been used to diagnose coagulopathy and guide hemostatic therapy in trauma. This systematic review of clinical studies in trauma investigates the ROTEM® parameters thresholds used for the diagnosing coagulopathy, predicting and guiding transfusion and predicting mortality.

**Methods:**

Systematic literature search was performed using MEDLINE, EMBASE and Cochrane databases. We included studies without restricting year of publication, language or geographic location. Original studies reporting the thresholds of ROTEM® parameters in the diagnosis or management of coagulopathy in trauma patients were included. Data on patient demographics, measures of coagulopathy, transfusion and mortality were extracted. We reported our findings according to the Preferred Reporting Items for Systematic Reviews and Meta-analyses (PRISMA) guidelines. Quality assessment and risk of bias were performed using Newcastle Ottawa Scale (NOS) and the quality assessment of diagnostic accuracy studies (QUADAS-2) tools, respectively.

**Results:**

A total of 13 observational studies involving 2835 adult trauma patients met the inclusion criteria. Nine studies were prospective and four were retrospective. There were no randomized controlled trials. The quality of the included studies was moderate (mean NOS 5.92, standard deviation 0.26). Using QUADAS-2, only 1 study (7.6 %) had low risk of bias in all domains, and 9 studies (69.2 %) had low risk of applicability concerns. Outcomes from 13 studies were grouped into three categories: diagnosis of coagulopathy (*n* = 10), prediction of massive transfusion or transfusion guidance (*n* = 6) and prediction of mortality (*n* = 6). Overall, specific ROTEM® parameters measured (clot amplitude and lysis) in the extrinsically activated test (EXTEM) and the fibrin-based extrinsically activated test (FIBTEM) were consistently associated with the diagnosis of coagulopathy, increased risk of bleeding and massive transfusion, and prediction of mortality. Presence of hyperfibrinolysis by ROTEM® was associated with increased mortality.

**Conclusions:**

Most of the evidence indicates that abnormal EXTEM and FIBTEM clot amplitude (CA5, CA10) or maximal clot firmness (MCF) diagnose coagulopathy, and predict blood transfusion and mortality. The presence of fibrinolysis (abnormal lysis index [LI30] or maximum lysis [ML]) was also associated with mortality. ROTEM® thus, may be of value in the early management of trauma patients.

**Electronic supplementary material:**

The online version of this article (doi:10.1186/s13049-016-0308-2) contains supplementary material, which is available to authorized users.

## Background

The degree of injury and hypoperfusion are implicated as initiators of the acute coagulopathy of trauma/shock (ACoTS) that occurs immediately after injury [1, 2]. ACoTS occurs in approximately 25 % of all severely injured patients and is associated with a three-fold increase in mortality [1]. Patients with ACoTS have complex coagulation defects, higher transfusion requirements, organ dysfunction, longer hospital stays and poorer outcomes including higher mortality rates [1, 3–5].

Current standards for the management of ACoTS are based mainly on results of standard coagulation tests (SCTs) [6–9] such as prothrombin time (PT), international normalized ratio (INR), activated partial thromboplastin time (aPTT), platelet count (PLT), and fibrinogen level [10]. Currently, many trauma centers use INR >1.5 and PLT <100 × 10^9^ L^−1^ to establish the presence of coagulopathy [11]. These values are widely used and incorporated into existing guidelines, despite lacking robust evidence support. [9, 11–13] Standard coagulation tests have long turnaround times (TAT), require transportation of the sample to the laboratory, separation of plasma from the red cells and detect only the initial phases of clot formation [7]. These tests were originally developed to diagnose coagulation abnormalities in congenital bleeding disorders and to monitor anticoagulation therapy, and their role in guiding transfusion therapy in trauma have not been validated [7]. Patients with ACoTS would benefit from a test that could quickly identify coagulation abnormalities, permit transfusion guidance, reduce exposure to allogeneic blood products and improve clinical outcomes by guiding rapid correction of any hemostatic defect. Thromboelastography (TEG®) and rotational thromboelastometry (ROTEM®) have been used in cardiovascular surgery and liver transplantation; [14–17] and, more recently, have been applied to trauma. The use of these devices may reduce hemorrhage as reported in a recent Cochrane systematic review [18] in patients requiring massive transfusion.

ROTEM® has recently been used as point-of-care (POC) test to optimize haemostatic resuscitation in trauma patients [19]. It utilizes a small volume of whole blood to assess hemostatic function from initiation of clot formation, clot propagation and clot lysis [20]. Additionally, ROTEM® provides information on platelet number/function and fibrinogen reserve. Luddington, in a review on ROTEM® assays and parameters, offers additional methodologic details about the test. [19] There are four assays which are run simultaneously: extrinsically activated test using tissue factor as activator (EXTEM) which detects defects associated with extrinsic pathway; intrinsically activated test using ellagic acid (INTEM) which detects defects of the intrinsic pathway; fibrinogen test (FIBTEM) using cytochalasin-D as platelet inhibitor which detects the contribution of fibrinogen to the clot; and a test using aprotinin inhibitor (APTEM), a test for hyperfibrinolysis (HF). In other clinical settings such as in liver transplantation, cardiac and vascular surgery, the use of ROTEM® has been linked to a reduction of exposure to allogeneic blood products and improvement of outcomes [16, 21–23]. In trauma, recent investigations suggest that ROTEM® can be used in the diagnosis of coagulopathy, prediction and guidance of transfusion and reduction of unnecessary exposure to allogeneic blood products [24–30]. Results of ROTEM® parameters are used for treatment decisions; however the reported thresholds used to diagnose coagulopathy and to guide transfusion vary substantially among reports.

There is a need for determining ROTEM® parameters and their thresholds that establish the presence of coagulopathy, predict bleeding, guide the hemostatic resuscitation and predict mortality. We conducted a systematic review of literature to summarize the reported ROTEM® parameters and their thresholds for this purpose. The primary goal was to determine evidenced based thresholds that could be incorporated into ROTEM® algorithms in the trauma resuscitation protocols.

## Methods

This descriptive systematic review was reported in accordance with Preferred Reporting Items for Systematic Reviews and Meta-Analyses (PRISMA) guidelines [31].

### Information sources and search technique

Two reviewers (PVV and LTDL) performed a systematic review of the indexed literature on ROTEM® studies that reported thresholds of ROTEM® parameters in trauma patients. The highly sensitive search strategy was developed by the review team in consultation with the health information specialist (Additional file 1). We searched MEDLINE, EMBASE and EBM Reviews (Cochrane Database of Systematic Reviews) from 1946 to March 2016 without restricting language or geographic location. The reviewers checked titles, abstracts, full texts, and personally contacted the authors (or manufacturer’s representative) to retrieve or clarify required information.

### Eligibility criteria and study selection

We searched for observational studies and randomized controlled trials (RCTs) in trauma where cut off values of ROTEM® parameters were reported in: (1) diagnosing coagulopathy; (2) predicting or guiding transfusion; and, (3) predicting mortality. We excluded animal studies, studies assessing patients with thermal injuries, case reports, case series involving <10 patients, and abstracts from conferences. Outcomes included accuracy in diagnosing coagulopathy, predicting massive transfusion, diminishing exposure to allogeneic blood products and predicting mortality. The reviewers independently screened titles and abstracts of all articles in a hierarchical manner by following the PRISMA guidelines [31, 32]. Titles were categorized as “include”, “exclude” or “undetermined” using an excel spreadsheet. The articles classified as “include” and “undetermined” by either reviewer were included for full text evaluation at the next level. Any discrepancies concerning agreement at both levels were resolved by discussion, consensus or consultation with a third reviewer (SR). Inter-rater agreement for inclusion was assessed using Cohen’s Kappa [33].

### Data abstraction and analysis

The reviewers independently collected data using standardized forms developed in collaboration with the study team. Reviewers were not blinded to the author or publication source of studies. The following data were collected: author, year, country, design, control group, patient population, duration of study, sample size, study objective, patient characteristics, and outcomes (coagulopathy, exposure to allogeneic blood products, and mortality). We also retrieved information regarding ROTEM® parameters, data on accuracy (sensitivity and specificity) of SCTs thresholds and ROTEM® thresholds used to diagnose coagulopathy, guide transfusion, predict exposure to allogeneic blood products, and predict mortality. The methodological quality of the studies was assessed using the Newcastle-Ottawa Scale [34] and Quality Assessment of Diagnostic Accuracy Studies-2 tool (QUADAS-2) [35] by both reviewers (PVV, LTDL).

## Results

**Fig. 1:**
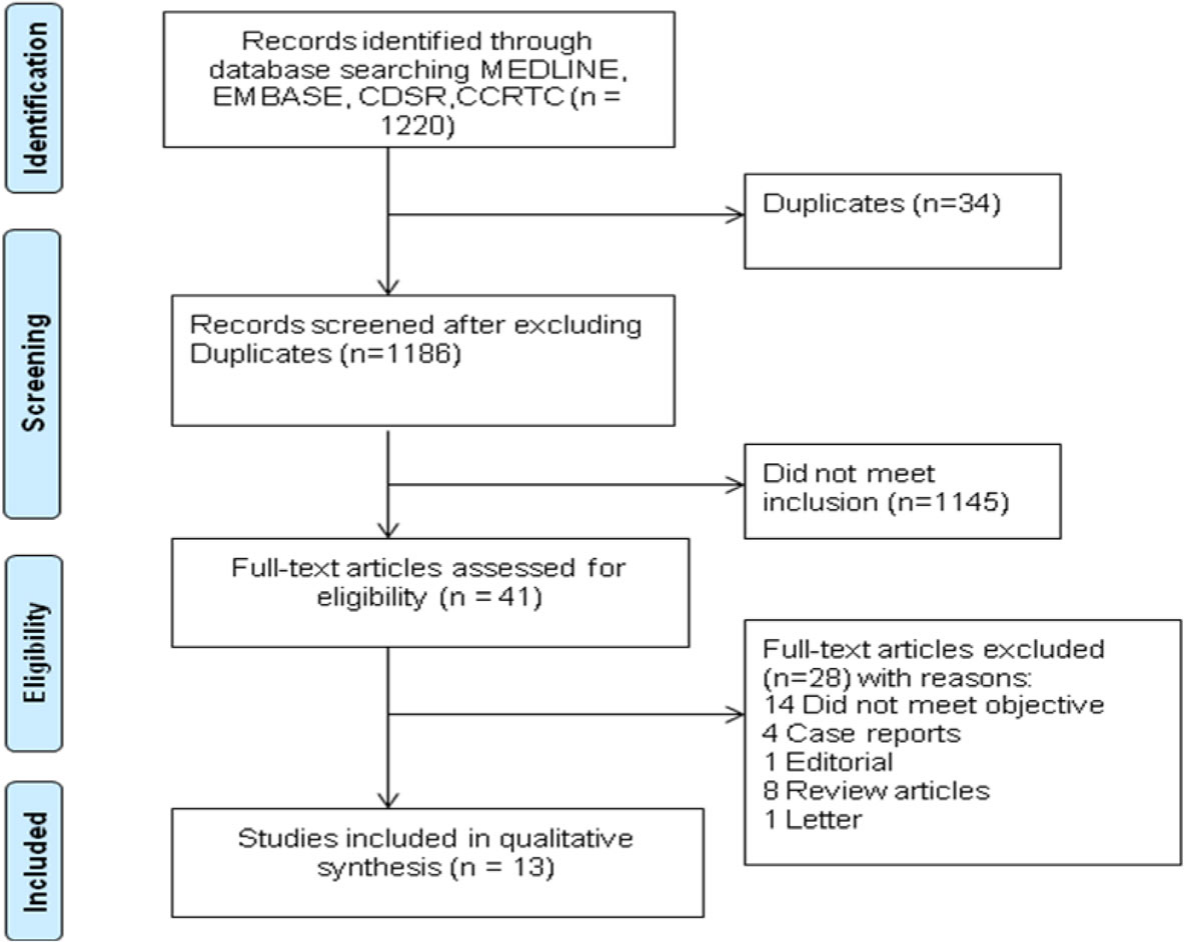
Flow Diagram of included and excluded studies

The search strategy identified a total of 1220 citations through MEDLINE, Cochrane and EMBASE data bases. We excluded 1179 citations as they were either unrelated to our question or were duplicates. Forty one citations were deemed relevant and were reviewed at full text level. Thirteen studies enrolling a total of 2835 patients met the eligibility criteria and were included (Fig. 1) [25–28, 30, 36–43]. We searched the references within each included study, and no additional studies were identified. The Kappa statistics for inter-rater agreement for titles plus abstracts, and full manuscript screening was 0.84 and 0.76, respectively.

### Study characteristics

Ten studies (Table 1) were conducted in Europe [26–28, 36–42]; two in Afghanistan, conducted by United Kingdom (UK) military researchers [25, 30] and one study included sites in both Europe and the UK [43]. All 13 studies were conducted in adult patients, included blunt and penetrating injuries, in the civilian [26–28, 36–43] or in the military settings [25, 30]. A single study included patients above 13 years of age [41] and no study was conducted exclusively in pediatric trauma patients. All but two [42, 43] studies were single centered [26–28, 36–41]. The sample sizes varied from 25 [25] to 808 [43] (median sample size 88, interquartile range 53–323). The median age of patients included in the studies ranged from 21 (IQR 18–35) [25] to 47 (IQR 26–66) [39] years. The percent of male patients ranged from 67 % [37] to 100 % [30]. All studies provided information on injury severity score (ISS), and the median ISS ranged from 12 (IQR 4–25) [41] to 75 (IQR 75–75) [37].

**Table 1:** Summary of studies included in the review

Author, region	Study design, centers, patients, years	*N*	Objective	ISS (mean ± SD or median/IQR)	Age (mean or median)	Sex male, *n* (%)
Rugeri, 2006 [36] France	Single center prospective, civilian, Jul 2004-Oct 2004	88	Detect coagulopathy Guide transfusion	22 (12–34)	34 (±16)	68 (77 %)
Levrat, 2008 [37] France	Single center prospective, civilian, Jul 2004-Oct 2004	87	Diagnosis of HF	Control group: 20 (11–29) HF group: 75 (75–75)	Control: 29 (21–43) HF: 30 (24–45)	Control: 64/82 (78 %) HF: 4/5 (80 %)
Schochl, 2009 [26] Austria	Single center prospective, civilian, Jan 2003-Dec 2007	33	Diagnosis of HF Predict mortality	47 ± 14	45 (20–88)	22 (67 %)
Doran, 2010 [25] Afghanistan	Single center prospective, military, Jan 2009-Mar 2009	25	Detect coagulopathy	MT group: 35 (25–50) Non MT: 20 (19–20)	21 (18–35)	25 (100 %)
Leemann, 2010 [38] Zurich	Single center retrospective, civilian, Jan 2006-Dec 2006	53	Predict MT	31.1 ± 1.7	39.6 (±2.5)	40 (75.5 %)
Schochl, 2010 [27] Austria	Single center retrospective, civilian, Jan 2005-Apr 2009	131	Guide transfusion	38 ± 15	46 ± 18	96 (73 %)
Tauber, 2011 [40] Austria	Single center, prospective, civilian, Jul 2005-Jul 2008	334	Detect coagulopathy Predict RBC transfusion Predict mortality	34 (24–45)	43 (27–56)	260 (77.8 %)
Schochl, 2011 [39] Austria	Single center, retrospective, civilian, Jan 2005-Oct 2010	88	Predict mortality	Survivors: 20 (16–26.25) Non survivors 29 (25–30.75)	47 (26–66)	67 (76 %)
Schochl, 2011 [28] Austria	Single center retrospective, civilian, Jan 2005-Dec 2010	323	Predict mortality	Non-MT group: 27 (20–34) MT group: 42 (34–50)	44 (26–59)	245 (78 %)
Davenport, 2011 [41] United Kingdom	Single center prospective, civilian, Jan 2007-Jun 2009	300	Detect coagulopathy Predict MT	12 (4–25)	33 (23–48)	246 (82 %)
Rourke, 2012 [42] England	Multicenter prospective, civilian, Jan 2008-Dec 2010	517	Detect coagulopathy Guide transfusion	14 (8–27)	36 (23–51)	405 (78 %)
Woolley, 2013 [30] Afghanistan	Single center prospective, military, May 2009-Jul 2009	48	Predict coagulopathy	34 (17–43)	24 (21–26)	48 (100 %)
Hagemo 2015 [43]	Multi center prospective civilian, Jan 2007-Nov2011	808	Detect coagulopathy, Predict MT	16 (20)	38 (28)	625 (77.4 %)

Legend: *FC* fibrinogen concentrate, *CA5* amplitude of the clot at 5 min, *CA10* amplitude of the clot at 10 min, *ISS* injury severity score, *HF* hyperfibrinolysis, *MCF* maximum clot firmness *MT* massive transfusion, *ROTEM*®® rotational thromboelastometry, *SLTs* standard laboratory tests, *TBI* traumatic brain injury

### Methodological quality

**Fig. 2:**
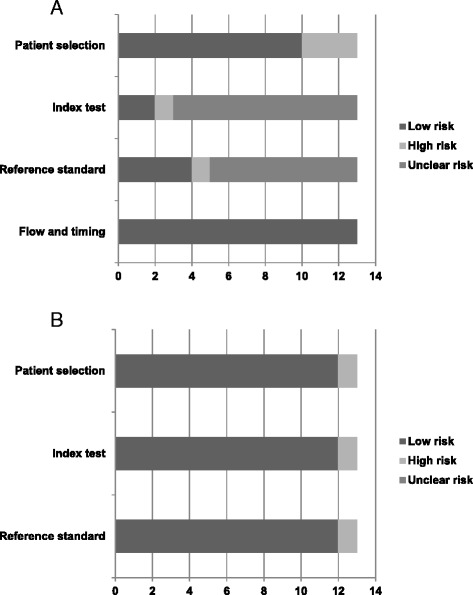
**a** Proportion of studies with low, high or unclear risks of bias. **b** Proportion of studies with low, high or unclear applicability concerns

There were no randomized controlled trials identified. Nine cohort studies were prospective [25, 26, 30, 36, 37, 40–43] and 4 were retrospective [27, 28, 38, 39]. The studies had moderate methodological quality as determined by the Newcastle Ottawa scale (NOS) (Table 2) with a mean score of 5.92 (SD = 0.26), with a possible range of 1 to 9. Ten studies enrolled consecutive patients [26–28, 36–42]. All studies had no comparable control group as defined in the NOS scale. Two studies used healthy volunteers as controls [30, 36] and one study used hospitalized polytrauma patients, and compared to patients with isolated brain injury [40]. All studies were assessed for quality of diagnostic accuracy using QUADAS-2 tool [35] (Table 3, Fig. 2a-b). Considering the domains of patient selection, index test, reference standard, flow and timing, only 1 study (7.6 %) had low risk of bias in all domains [40]; 7 studies (53.8 %) had low and unclear risks [26, 28, 30, 38, 39, 41, 42]; and 5 studies (38.4 %) had high risk of bias in at least 1 domain [25, 27, 36, 37, 43]. In terms of applicability concerns, 9 studies (69.2 %) [26–28, 30, 38, 39, 41–43] had low concerns and 4 studies (30.7 %) [25, 36, 37, 40] had at least 1 domain with high concern.

**Table 2:** The Newcastle Ottawa scale for the cohort studies included in the review

Reference	Representativeness of the exposed cohort	Selection of non-exposed cohort	Ascertainment of exposure	Outcome not present at start	Comparability of controls	Assessment of outcome	Adequate follow up	Loss to follow up	Total score
Rugeri 2007 [36]	*	-	*	*	-	*	*	*	6/9
Levrat 2008 [37]	*	-	*	*	-	*	*	*	6/9
Schöchl 2009 [26]	*	-	*	*	-	*	*	*	6/9
Doran 2010 [25]	*	-	*	*	-	*	*	-	5/9
Leemann 2010 [38]	*	-	*	*	-	*	*	*	6/9
Schochl 2010 [27]	*	-	*	*	-	*	*	*	6/9
Tauber 2011 [40]	*	-	*	*	-	*	*	*	6/9
Schochl 2010 [39]	*	-	*	*	-	*	*	*	6/9
Davenport 2011 [41]	*	-	*	*	-	*	*	*	6/9
Schöchl 2011 [28]	*	-	*	*	-	*	*	*	6/9
Rourke 2012 [42]	*	-	*	*	-	*	*	*	6/9
Woolley 2012 [30]	*	-	*	*	-	*	*	*	6/9
Hagemo 2015 [43]	*	-	*	*	-	*	*	*	6/9

Legend: Refer to http://www.ohri.ca/programs/clinical_epidemiology/oxford.asp, for a description of Newcastle-Ottawa Quality Assessment Scale for cohort studies. In general, more stars denote higher quality. ‘Representativeness’ is awarded a star if the cohort is truly or somewhat representative of the population of interest. For selection of the non-exposed cohort, a star is awarded if it is drawn from the same population as the exposed cohort. The relevant exposure in this review is management using ROTEM®; we considered a non-exposed cohort to be one that was managed without ROTEM®; other studies used healthy or other hospitalized controls to examine associations between ROTEM® abnormalities and outcomes [30, 36, 40]. Exposure is satisfactorily ascertained if data are collected from a secure record. A star is awarded if the outcome is not present at the start of the study. A maximum of two stars can be given for ‘Comparability of controls’ for controlling of confounders in either the design (matching) or analysis (statistical adjustment) phase. We also gave one star when selection criteria appeared to create comparable groups via restriction. ‘Assessment of outcome’ is awarded a star if the outcomes were assessed by independent blind assessment or record linkage; we also considered the outcome of mortality to be adequately assessed in all studies reporting it due to low risk of bias. The duration of follow-up was considered adequate if it was long enough for the outcomes to occur. Completeness of follow-up was considered adequate if all patients were accounted for or if the number lost to follow-up was sufficiently low to be unlikely to introduce bias

**Table 3:** QUADAS-2 Tool: summary of assessment of risk of bias and applicability concerns

Risk of bias					Applicability concerns		
Reference	Patient selection	Index test	Reference standard	Flow and timing	Patient selection	Index test	Reference standard
Rugeri 2007 [36]	☺	☹	☺	☺	☺	☹	☺
Levrat 2008 [37]	☺	?	☹	☺	☺	☺	☹
Schöchl 2009 [26]	☺	?	?	☺	☺	☺	☺
Doran 2010 [25]	☹	?	?	☺	☹	☺	☺
Leemann 2010 [38]	☺	?	?	☺	☺	☺	☺
Schochl 2010 [27]	☹	☺	☺	☺	☺	☺	☺
Schochl 2011 [39]	☺	?	?	☺	☺	☺	☺
Davenport 2011 [41]	☺	?	?	☺	☺	☺	☺
Schöchl 2011 [28]	☺	?	?	☺	☺	☺	☺
Tauber 2011 [40]	☺	☺	☺	☺	☹	☺	☺
Rourke 2012 [42]	☺	?	?	☺	☺	☺	☺
Woolley 2012 [30]	☺	?	☺	☺	☺	☺	☺
Hagemo 2015 [43]	☹	?	?	☺	☺	☺	☺

Legend: ☹ denotes high risk of bias, ☺ denotes low risk of bias, and ? denotes unclear risk of bias

### Outcomes

We found ten studies addressing ROTEM® thresholds for diagnosis of coagulopathy [25, 26, 30, 36, 37, 39–43], 6 studies addressing thresholds for prediction or guidance of transfusion [27, 28, 38, 40, 41, 43], and 6 studies addressing prediction of mortality [26, 27, 37, 39, 40, 42]. Two studies used 4 ROTEM® assays (EXTEM, INTEM, FIBTEM and APTEM) [23, 33]; 4 studies used 3 assays (EXTEM, INTEM, and FIBTEM) [25, 28, 36, 39]; 4 studies used 2 assays (EXTEM and FIBTEM) [27, 40, 42, 43]; 1 study used 2 other assays (EXTEM and INTEM) [38] and 2 studies used 1 assay (EXTEM) [30, 41].

### Studies addressing thresholds of ROTEM® parameters to diagnose ACoTS

Definition of coagulopathy by SCTs and ROTEM® parameters varied across all studies. Five studies [25, 36, 39, 41, 43] used different SCTs as gold standards, with different cut off values to define coagulopathy. For example, one study used INR >1.6 and/or aPTT >60s and/or a platelet count <100 × 10^9^ L^−1^ and/or fibrinogen < 1 g/L [36]. In contrast, another study used a prothrombin time index (PTI) test <70 % (a value of <70 % in PTI is equivalent to INR >1.3), aPTT >35 s, and fibrinogen <1.50 g/L [39]. Lastly, coagulopathy was defined by Davenport and Hagemo as an INR >1.2 [41, 43].

In ROTEM®, 10 studies reported thresholds of parameters in detecting the various defects in ACoTS [25, 26, 30, 36, 37, 39–43] (Table 4). However, there was a wide variation on the parameters chosen, and their cut off values. Better designed studies used SCTs as controls when determining thresholds and cut-off values. Other studies used the recommendations from previous consensus meetings [44]. Finally, in other studies, authors used previous institutional experience, or cut-off values pre-established by the ROTEM® manufacturer. Studies that used SCTs as reference standards for comparisons are described here. Table 4 describes all evidence in details.

**Table 4:** Studies addressing ROTEM® thresholds used for diagnosis of trauma coagulopathies

Study	Comparator	ROTEM® thresholds used	Accuracy of threshold Sensitivity/Specificity		AUC	Key findings
Rugeri 2006 [36]	PTR > 1.5 FIB < 1.0 g/L	EXTEM CA15 = 32 mm FIBTEM A10 = 5 mm	87 91	100 85	0.98 0.96	1 – Significant correlation between EXTEM CA15 < 32 mm and PT >1.5 (r = 0.66, p < 0.0001) and of FIBTEM CA10 < 5 mm and Fibrinogen <1.0 g/L (*r* = 0.85, *p* < 0.0001). 2 – EXTEM A15 = 32 and FIBTEM A10 = 5 mm had a higher sensitivity and specificity to detect PTr > 1.5 and fibrinogen <1.0 g/L
Levrat 2008 [37]	ELT < 90s	EXTEM MCF ≤ 18 mm LI30 ≤ 71 % APTEM MCF ↑by 7 %	100 75 80	100 100 100	1.00 0.87 0.92	1 – MCF correlated well with ELT when compared with amplitude and CLI. 2 – HF patients exhibited greater ROTEM® abnormalities, lower INR, lower fibrinogen levels and were more severely injured (↑ ISS) compared to the control group (all *p* < 0.05)
Schochl 2009 [26]	No comparator	EXTEM and INTEM ML = 100 %	NA	NA	NA	1 – Fulminant HF confirmed by complete clot lysis within 30 min by ROTEM® trace
Doran 2010 [25]	PT > 18 s aPTT > 38 s	EXTEM MCF < 45 mm	NA	NA	NA	1 – ROTEM® detected coagulation abnormalities in 64 % patients vs. 10 % detected by SCTs as compared to test reference ranges? (*p* = 0.0005). 2 – MCF < 45 mm in 100 % of MT patients
Davenport 2011 [41]	PTR > 1.2	EXTEM CA5 ≤ 35 mm	77	NA	NA	1 – EXTEM CA5 ≤ 35 mm detected coagulopathy with 77 % sensitivity and a false positive rate of 13 %
Tauber, 2011 [40]	INR > 1.5 aPTT > 50s	EXTEM MCF < 45 mm	72	76	0.83	1 - Prevalence of low fibrinogen, impaired fibrin polymerization and reduced MCF was 26 %, 30 %, and 22 %, respectively, higher than the prolonged INR (14 %) 2 – There was ↑ F1 + 2 and TAT and low AT levels, indicating ↑ thrombin formation among all patients
	FIB < 1.5 g/L PLT < 100 × 10^3^	FIBTEM MCF < 7 mm LI60 < 85 %	86 79	71 78	0.89 0.84	
Schochl, 2011 [39]	PTI < 70 %,	EXTEM CT > 80s CFT > 159 s MCF < 50 mm	NA	NA	0.77	1 – Coagulopathy was characterized by abnormal values in most or all ROTEM® measurements as compared to reference range vs. SCT. 2 – Significantly low CA5-CA30 min, MCF in EXTEM, INTEM and FIBTEM assays in non survivors vs. survivors (*p* < 0.01)
	aPTT > 35 s, PLT < 100 × 10^3^	INTEM CT > 240 s CFT > 110 s MCF < 50 mm				
	FIB < 1.5 g/L	FIBTEM MCF < 9 mm				
Rourke 2012 [42]	FIB < 1.5 g/L	EXTEM CA5 < 36 mm FIBTEM CA5 < 9.5 mm	53 78	87 70	NA NA	1 – ROTEM® parameters correlated with fibrinogen level. 2 – Ex vivo fibrinogen administration reversed coagulopathy by ROTEM®.
Woolley 2012 [30]	PT > 1.5	EXTEM CA5 < 32 mm EXTEM A10 < 40 mm	96 100	58 70	NA NA	1 – EXTEM MCF < 40 mm and interim values of EXTEM A5 and A10 predicted coagulopathy (A15: sensitivity/specificity of 96 %/58 % and for A10: sensitivity/specificity 100 %/70 %)
Hagemo 2015 [43]	INR > 1.2	EXTEM CA5 < 37 mm FIBTEM CA5 < 8 mm	NA NA	NA NA	0.79 0.80	1 – Highest ROTEM® AUC values were found for EXTEM CA5 and FIBTEM CA5 for detecting ACoTS 2 – EXTEM CA5 ≤ 37 mm had a detection rate of 66.3 % and FIBTEM CA ≤ 8 mm had a detection rate of 67.5 % of ACoTS

Legend: *aPTT* activated partial thromboplastin time, *AT* antithrombin III, *AUC* area under curve, *CA* clot amplitude (measured at 5,10,15 min, etc.), *CFT* clot formation time, *ER* emergency room, *ED* Emergency department, *ELT* euglobin lysis time, *F1* + *2* prothrombin complex, *FC* fibrinogen concentrate, *EXTEM* extrinsically activated test with tissue factor, *FDP* fibrin degradation products, *FIB* fibrinogen, *FIBTEM* fibrin-based extrinsically activated test with tissue factor and the platelet inhibitor cytochalasin D, *GCS* Glasgow Coma Scale, *HF* hyperfibrinolysis, *HGB* hemoglobin, *INR* international normalized ratio, *HCT* hematocrit, *ISS* injury severity score, *LI30* lysis index at 30 min, *MCF* maximum clot firmness, *ML* maximum lysis, *mm* millimeter, *MT* massive transfusion, *NA* not available, *NPV* negative predictive value, *OR* operation room, *PLT* platelet, *PPV* positive predictive value, *PT* prothrombin time, *PTR* prothrombin ratio, *s* seconds, *sen* sensitivity, *SLTs* standard laboratory tests, *spec* specificity, *TAT* thrombin antithrombin complex, *TBI* traumatic brain injury

#### ROTEM® thresholds determined with comparison to standard controls (SCTs)

In a study conducted by Rugeri [36], thresholds were determined by evaluating the extent of correlation between ROTEM® parameters with corresponding SCTs (CA15-EXTEM with PT: *r* = 0.66, *p* < 0.0001); clot formation time [CFT]-INTEM with aPTT: *r* = 0.91, *p* < 0.0001; CA10-FIBTEM with fibrinogen level: *r* = 0.85, *p* < 0.0001; CA15-INTEM with PLT count: *r* = 0.57, *p* < 0.0001). The group found cut-off values of EXTEM CA15 < 32 mm and FIBTEM CA10 < 5 mm to detect laboratory PT > 1.5 and fibrinogen level <1 g/L, with high sensitivity (87 % and 91 %) and specificity (100 and 85 %), respectively. In another study, Levrat determined the cut off values by assessing correlation between ROTEM® parameters and euglobin lysis time (ELT), used as the gold standard control [37]. In this study, a threshold of 18 mm (MCF-EXTEM), 71 % (CLI30) AND 7 % increase of MCF-APTEM, sensitivity was, 100, 75 and 80 %, respectively with a specificity of 100 %. Moreover, Davenport [41] and Rourke [42] used ROTEM® CA5 < 35 mm as threshold based on correlation with normal PT values to discriminate normal from the abnormal curves in patients with ACoTS. Hagemo used ROTEM® threshold value of EXTEM CA5 ≤ 37 mm and FIBTEM A5 ≤ 8 mm to detect ACoTS [43]. These authors used INR > 1.2 and fibrinogen concentration of ≤1.61 g/L to define ACoTS, respectively [43]. A feasibility study [25] in a deployed military trauma setting demonstrated that an abnormal CA10 was associated with a subsequent development of an abnormal MCF (<45 mm). MCF <45 mm was present in a 100 % of MT patients. When PT > 18 s and aPTT >60s were used as the gold standard for ACoTS, only 10.5 % of patients were defined as coagulopathic. By comparing these results with ROTEM® results, it was found that 64 % were coagulopathic (FIBTEM-MCF < 45 mm), (*p* = 0.0005). Another study [41] reported that the threshold EXTEM CA5 ≤ 35 mm predicted INR > 1.2 in 77 % of cases. In TBI patients, Schochl [39] reported a cut off value of EXTEM-CT > 80s, compared to PTI < 70 %, to define coagulopathy (*p* = 0.003). Finally, in another study in the military setting [30] the authors compared PT > 18 s (gold standard) to diagnose coagulopathy and identified that early CA5 < 32 mm and CA10 < 40 mm predicted the hypocoagulation state with a sensitivity/specificity of CA5 96/58 % and CA10 100/70 %, respectively, compared to SCTs.

#### Hypofibrinogenemia

Two studies investigated the use of FIBTEM CA10 < 5 mm and FIBTEM CA5 < 10 mm for diagnosing different degrees of hypofibrinogenemia. In the first study [36], FIBTEM CA10 < 5 mm diagnosed fibrinogen levels below 1.0 g/L with sensitivity of 87 % and specificity of 91 %. The second study [42] reported EXTEM CA5 < 36 mm with a sensitivity of 53 % and specificity of 87 % for discerning patients with fibrinogen levels <1.5 g/L. For FIBTEM CA5 < 10 mm, the reported sensitivity and specificity were 78 and 70 % respectively for predicting a fibrinogen level below 1.5 g/L.

#### Hyperfibrinolysis

Three studies [26, 37, 40] reported thresholds of different ROTEM® parameters to diagnose degrees of hyperfibrinolysis (HF) such as mild, moderate and fulminant. Two studies [26, 37] compared their findings with SCTs. The first study [37] defined HF as euglobulin lysis time (ELT) <90 min (used as gold standard) in a series of 23 patients. The authors used EXTEM MCF ≤ 18 mm, clot lysis index at 30 min (CLI30) < 71 % and APTEM MCF 7 % increase to define hyperfibrinolysis (HF) (sensitivity 100, 75, 80 % and specificity 100 % for all, respectively) in these patients with an abnormal ELT test. The second study [26] enrolled 33 trauma patients diagnosed with HF by ROTEM®. They used clot lysis in EXTEM and INTEM assays at different time points across the ROTEM® tracing to define the three patterns of HF, confirmed by the APTEM test. A complete clot lysis (ML = 100 %) within 30 min was used to define patients with fulminant HF; complete clot lysis between 30 and 60 min defined intermediate HF and complete clot lysis after 1 h to define late HF. The median values of laboratory fibrinogen was lower in fulminant HF group and intermediate HF group when compared with late HF group (fulminant HF: 0.5 g/L; intermediate HF: 0.49 g/L compared with late HF 1.04 g/L, *p* = 0.048 for both).

#### Platelet count

A single study [36] evaluated the correlation between platelet count and INTEM CA15 (*r* = 0.57, *p* < 0.0001). However, the threshold value of INTEM CA15 = 46 mm showed poor positive predictive (PPV) values in the diagnosis of laboratory platelet count below 50 × 10^−9^L^−1^ (sensitivity: 100 % [95 % CI 71–100], specificity 83 % [95 % CI 82–83]; PPV 17 % [95 % CI 12–17], negative predictive value [NPV] 100 % [95 % CI 98–100]; AUC 0.92).

### Studies addressing thresholds of ROTEM® parameters in predicting or guiding transfusion

#### Predicting transfusion

Six studies reported ROTEM® thresholds either in predicting transfusion [28, 38, 40, 41, 43], including MT [28, 38, 41, 43], or guiding transfusion [27] (Table 5). Massive transfusion was defined by the need for transfusion of ≥10U of RBCs within the first 12 h [41] or 24 h [28, 38] of hospital admission in three studies. Values outside the reference range for EXTEM and INTEM CT, CFT, CA at 10, 20 and 30 min, as well as reduced MCF were more likely in patients who required a MT vs. patients who did not (*p* < 0.0001, for all) [28, 38]. The reference ranges used in this study were the same established by the same group, in a previous study that used SCTs as control [26].

**Table 5:** Studies addressing ROTEM® thresholds used to predict or guide blood transfusion

Study	Comparator	ROTEM® thresholds used	Accuracy of threshold Sensitivity/Specificity		ROC/AUC	Key findings
Massive transfusion						
Leemann 2010 [38]	aPTT > 36 s PLT < 100 × 10^3^ INR > 1.2	EXTEM/INTEM CA10, CA20, CFT, MCF as per manufacturer	NA	NA	0.82	2 – INTEM MCF 37.5 ± 2.9 associated with MT requirements within 24 h
Tauber, 2011 [40]	FIB 1.50 g/L INR > 1.5	FIBTEM MCF < 7 mm	71	NA	0.80	1 – FIBTEM MCF < 7 mm associated with RBC use (OR 0.92, 95 % CI 0.87–0.98)
Schochl 2011 [28]	PLT ≤ 161 × 10^3^ aPTT ≤ 35.2 s FIB ≤ 1.4 g/dL	FIBTEM A10 ≤ 4 mm FIBTEM MCF ≤ 7 mm	63.3 77.5	83.2 74.9	0.83 0.84	1 – 85 % patients with FIBTEM MCF 0–3 mm received MT 2 – FIBTEM A10 (0.83) and FIBTEM MCF (0.84) showed high predictive value for MT
Davenport 2011 [41]	PTR > 1.2	EXTEM CA5 ≤ 35 mm	71.4 %	NA	NA	1 – CA5 identified patients who required MT with detection rate of 71 % vs. 43 % for PTR > 1.2, *p* < 0.001
Hagemo 2015 [43]	INR > 1.2	EXTEM CA5 ≤ 40 mm FIBTEM CA5 ≤ 9 mm	72.7 %	77.5 %	0.75 0.78	1 – ROTEM CA5 is a valid predictor for MT.
Any transfusion						
Schochl, 2010 [27]	PT (11–13.5 s) aPTT (26-35 s) FIB (2–4.5 g/L) PLT (150–350)	FIBTEM MCF < 10 mm EXTEM CT > 1.5× normal	NA	NA	NA	1 – ROTEM® guided FC and PCC transfusion, associated with favorable survival (24.4 % vs. 33.7 %; *p* = 0.032)
Davenport 2011 [41]	PTR > 1.2	EXTEM CA5 ≤ 35 mm CT > 94 s Alpha < 65^0^	33.3 %	NA	NA	1 – CA5 ≤ 35 mm predicted RBC and plasma transfusion. Patients with CA5 ≤ 35 mm received more RBC (46 % vs. 17 %, *p* < 0.001) and plasma (37 % vs. 11 %, *p* < 0.001) transfusions. 2 – CA5 ≤ 35 mm received more RBC (4U vs. 1U, *p* < 0.001) and FFP (2U vs. 0U, *p* < 0.001)

Legend: *aPTT* activated partial thromboplastin time, *CA* clot amplitude (measured at 5, 10, 15, 20 min, etc.), *CT* clotting time, *CFT* clot formation time, *ED* emergency department, *EXTEM* extrinsically activated test with tissue factor; *FIB* fibrinogen, *FC* fibrinogen concentrate, *FFP* fresh frozen plasma, *FIBTEM* fibrin-based extrinsically activated test with tissue factor and the platelet inhibitor cytochalasin D, *GCS* Glasgow coma scale, *HGB* hemoglobin, *INR* international normalized ratio, *ISS* injury severity score, *MCF* maximum clot firmness, *MT* massive transfusion, *NA* not available, *PC* platelet concentrate, *PCC* prothrombin complex concentrate, *PLT* platelets, *RBC* red blood cells, *PT* prothrombin time, *PTI* prothrombin time index, *PTR* prothrombin time ratio, *RBC* red blood cells

Davenport [41] demonstrated that EXTEM CA5 ≤ 35 mm predicted the need for MT with higher detection rate compared to INR > 1.2 (71 vs. 43 %, *p* < 0.001). Schochl [28], using threshold pre-established in a previous study by the same group [26] reported that both FIBTEM A10 ≤ 4 mm (ROC AUC = 0.83) and FIBTEM MCF ≤ 7 mm (ROC AUC = 0.84) were predictive of the need for MT. Lastly, Hagemo [43] demonstrated that threshold values of EXTEM CA5 ≤ 40 mm predicted MT in 72.7 % and FIBTEM CA5 ≤ 9 mm predicted MT in 77.5 %, respectively. However detection rate for MT was found to be highest for INR, as compared to EXTEM CA5 (51.1 and 45.5 %, respectively). The optimum threshold value for fibrinogen in predicting MT was ≤1.90 g/L with a detection rate of 77.8 % and a positive predictive value of 14.

#### Guiding transfusion

Schochl [27], in a retrospective analysis of trauma patients who received ≥5U RBCs within 24 h, and whose coagulation management was guided by ROTEM®, developed a clinical practice guideline using thresholds of ROTEM® parameters to guide transfusion. The group used a threshold of FIBTEM MCF < 10 mm to guide transfusion of fibrinogen concentrate (FC) and used EXTEM CT > 1.5 times normal to guide PCC administration. Reference ranges used for these ROTEM® tests’ parameters were previously determined in a multi-center investigation by Lang [45]. The authors were able to demonstrate a reduction in the number of RBC units transfused. The use of RBC units was avoided in 29 % of patients receiving FC and PCC therapy compared to only 3 % avoided in the group receiving fresh frozen plasma (FFP) (*p* < 0.001).

#### Studies addressing thresholds of ROTEM® parameters in predicting mortality

Six studies evaluated ROTEM® thresholds in predicting mortality (Table 6) [26, 27, 37, 39, 40, 42]. These studies evaluated mortality at different time points, including: within 24 h of arrival [40]; death in hospital [26, 40]; death within 24 h and 28 days [42], 30 days [40], and two studies did not define the time to death [27, 37]. Two studies reported that trauma patients with the diagnosis of HF had higher rates of mortality [26, 37]. The studies defined HF differently: Schochl defined HF as a complete clot lysis (ML = 100 %) on ROTEM® at different time intervals as fulminant HF, intermediate HF and late HF as described above [26]. Finally, Levrat defined HF as an ELT < 90 min. [37] We describe here under the studies that adjusted their findings for confounders, or compared findings with previously validated trauma scores [46, 47]. The full description of the evidence is on Table 6.

**Table 6:** Studies addressing ROTEM®® thresholds for the prediction/reduction of mortality

Study	Comparator	Optimal ROTEM®® Parameter and cut off	Accuracy of threshold			Key findings
			Sensitivity	Specificity	AUC	
Levrat 2008 [37]	ELT < 90 min	EXTEM MCF ≤ 18 mm LI30 ≤ 71 % APTEM MCF ↑ by 7 %	100 75 80	100 100 100	1.00 0.87 0.80	1 – Patients with HF had higher mortality rate (100 %, CI: 48–100 % vs. 11 % CI: 5–20 %, *p* < 0.05)
Schochl 2009 [26]	No comparator	ML = 100 %	NA	NA	NA	1 – Fulminant HF associated with 100 % mortality 2 – ↑CFT and ↓PLT contribution to MCF associated with ↑mortality (*p* = 0.042 and *p* = 0.026 respectively)
Schochl 2010 [27]	No comparator	FIBTEM MCF < 10 mm EXTEM CT > 1.5 × normal	NA	NA	NA	1 – Observed mortality was lower than the predicted mortality by TRISS (24.4 % vs.33.7 %, *p* = 0.032) with a favourable survival rate.
Tauber, 2011 [40]	PT = 70 % FIB = 1.82 g/L	FIBTEM MCF < 7 mm, EXTEM CT 91 s EXTEM CFT 218 s EXTEM MCF 46 mm	NA	NA	0.8	1 – FIBTEM MCF < 7 mm and EXTEM MCF < 45 mm associated with higher mortality (21 % vs. 9 % SCTs, *p* = 0.006 and 25.4 % vs. 9.4 % SCTs, *p* < 0.001, respectively) 2 – EXTEM MCF had strong association with early deaths (OR 0.94, 95 % CI 0.9–0.99).
Schochl, 2011 [39]	aPTT > 35 s	FIBTEM MCF < 9 mm	NA	NA	0.77	1 – Decrease in clotting times in EXTEM and INTEM (*p* < 0.001), decreased CFT in EXTEM and INTEM (*p* < 0.0001), and increased MCF in EXTEM, INTEM, and FIBTEM (*p* < 0.01) were noted in survivors compared with non-survivors, in patients with severe isolated TBI 2 – FIBTEM MCF (ROC 0.77, 95 % CI 0.66.5–0.85, *p* < 0.001) and aPTT (ROC 0.79 95 % CI 0.68–0.86, *p* < 0.001) independently associated with mortality.
Rourke, 2012 [42]	FIB < 1.5 g/L	EXTEM CA5 < 36 mm FIBTEM CA5 < 10 mm	53 78	87 70	NA NA	1 – Fibrinogen level was independently associated with higher mortality at 24 h and 28 days (*p* < 0.001). ROTEM could detect hypofibrinogenemia early and rapid replacement of fibrinogen may improve outcomes.

Legend: *aPTT* activated partial thromboplastin time, *APTEM* EXTEM test inactivated using aprotinin, *CA5* clot amplitude at 5 min, *CT* clotting time, *CFT* clot formation time, *ELT* euglobulin lysis time, *EXTEM* extrinsically activated test with tissue factor, *FIB* fibrinogen, *FIBTEM* fibrin-based extrinsically activated test with tissue factor and the platelet inhibitor cytochalasin D, *HF* hyperfibrinolysis, *INTEM* intrinsically activated test, *LI30* lysis index at 30 min, *MCF* maximum clot firmness, *ML* maximum lysis, *NA* not available, *OR* odds ratio, *PC* platelet concentrate, *ROC* receiver operating curve, *s* seconds, *SCTs* standard coagulation tests, *TRISS* Trauma injury severity score

Tauber [40] found a significant increase in mortality with FIBTEM < 7 mm (21 vs. 9 %, *p* = 0.006) and EXTEM MCF < 45 mm (25.4 vs 9.4 %, *p* < 0.001). Similarly, EXTEM MCF was independently and negatively associated with early mortality (OR 0.94, 95 % CI 0.9–0.99). The author additionally reported 85.7 % mortality in patients with fulminant HF (ML100% within 30 min), and 11.1 % mortality in patients with moderate HF (ML100% between 30 and 60 min). Rourke [42] reported that a low FIBTEM A5 < 9.5 mm was an independent predictor of 24 h and 28 days mortality (*p* < 0.001).

In a study in brain injury patients, Schochl [39] demonstrated an independent association between FIBTEM MCF < 9 mm (ROC: 0.77; 95 % CI, 0.665–0.850, *p* < 0.001) and aPTT > 35 s (ROC 0.79; 95 % CI 0.686–0.868, *p* < 0.001), and mortality. Moreover, in this study, ROTEM® revealed shorter CT in EXTEM and INTEM (*p* < 0.001), shorter CFT in EXTEM and INTEM (*p* < 0.0001), and higher MCF in EXTEM and INTEM (*p* < 0.01) in survivors compared with non-survivors. Finally, in another study conducted the Schochl [27], where trauma patient resuscitation was guided by ROTEM® with FC and PCC, a reduction in the observed mortality than the predicted mortality by TRISS and RSS was demonstrated (24.4 vs. 33.7 %, *p* = 0.032).

## Discussion

### Main findings

We performed a systematic review of the literature to ascertain the existing evidence on the reported thresholds of ROTEM® parameters in diagnosing coagulopathy, predicting or guiding transfusion and predicting mortality in trauma patients. Thirteen studies evaluating 2835 patients met our inclusion criteria. Overall, the methodological quality of the included studies was moderate. In general, the patient populations were different across studies. We found studies using different ROTEM® parameters and different thresholds for the same ROTEM® parameter for the diagnosis of coagulopathy and guidance of component transfusions. We did not find any randomized controlled trials, and the majority of the retrospective or prospective cohort studies found in the review did not have a gold standard coagulation test used for comparison. Where SCTs results were used as the gold standard, there were no studies done to validate the chosen cut-off. We were not able to pool the data and conduct meta-analysis due to the marked clinical heterogeneity among the studies. Considering the limited number of studies and the moderate methodological quality, we concluded that there is still no robust evidence supporting the thresholds of ROTEM® parameters reported in the literature in diagnosing coagulopathy, guiding or predicting transfusion, and predicting mortality.

For diagnosis of coagulopathy, the most properly designed studies, using a control (SCTs), identified several different parameters and thresholds. However, definition of coagulopathy by SCTs was not standardized. Most common parameters used to define coagulopathy across the studies were EXTEM-CA5, CA10, CA15, which were correlated with PT and INR. The cut-off values varied from 5 mm in CA5 to 35 mm in CA15. Of note, several other studies used arbitrary values obtained from previous expert group meetings, previous author’s experience or from reference values from the manufacturer.

Hypofibrinogenemia was diagnosed, in general, with FIBTEM CA5 and CA10 (<10 mm and <5 mm, respectively). Gold standards SCTs used for comparison were fibrinogen <1.0 g/L or 1.5 g/L. In the assessment of hyperfibrinolysis, complete clot lysis (ML100 %) and LI60 < 85 % were used as definition of hyperfibrinolysis, with the ELT < 90 min as the gold standard for comparison.

For prediction of transfusion, the best designed study established EXTEM-CA5 ≤ 35 mm using INR as control. The other studies did not use SCTs as gold standards for control. In those studies, the parameters and their cut off values were established from previous author’s experiences or from the manufacturer of the ROTEM® device. Values outside the reference range for EXTEM and INTEM CT, CFT, CA at 10, 20 and 30 min, as well as reduced MCF were more likely in patients who required a MT, as compared to patients who did not. Other parameters used were EXTEM CA5 ≤ 35 mm and FIBTEM-MCF ≤ 7 mm that were also associated with the need for MT.

In transfusion guidance, FIBTEM MCF < 10 mm and EXTEM CT > 1.5 times normal were used to guide administration of FC and PCC, respectively, with a reduction of the number of RBC units used in the FC/PCC group, compared to fresh frozen plasma (FFP) group (*p* < 0.001). No other study reported on ROTEM® metrics utilized to guide transfusion.

Mortality was assessed in different studies, and overall, an association between hyperfibrinolysis and mortality was demonstrated (maximum lysis of 100 %, defined using ELT as control). Multiple parameters were found to be associated with mortality, including: FIBTEM < 7 mm/<9 mm/<9.5 mm, EXTEM-MCF < 45 mm; shorter EXTEM-CT, INTEM-CT, EXTEM-CFT and INTEM-CFT; higher EXTEM-MCF, INTEM-MCF.

Two systematic reviews of ROTEM® and TEG®, the similar viscoelastic currently mostly used in United States, exist for nontrauma populations. A Cochrane review [18] included nine RCTs, mostly in cardiac surgery, that compared transfusion guided by ROTEM® and TEG® with transfusion guided by clinical judgment, SCTs, or both in severely bleeding patients. This review found that ROTEM® and TEG® reduced blood loss by a mean of 85 ml (95 % CI, 29 to 141 ml) but had no effect on mortality. Another systematic review [48] included 16 observational studies and two RCTs in patients with sepsis and concluded that ROTEM® and TEG® (compared with SCTs) may detect impaired fibrinolysis, which may help to discriminate between sepsis and systemic inflammatory response syndrome (SIRS). Aside from current moderate quality, the evolving trauma literature brings evidence that ROTEM® has the potential to diagnose ACoTS, and predict and guide transfusion faster than the SCTs due to the point of care nature of the tests. Cut-off values of various ROTEM® parameters may diagnose the different nuances of ACoTS such as the different causes for hypocoagulation (low levels of clotting factors, fibrinogen and platelets, and platelet dysfunction), and hyperfibrinolysis.

### Strengths and weaknesses of this study, and future research

Major limitations of this review are related to the quality of the included studies, which were not powered with proper sample sizes for detection of differences, for example. The studies were only observational, and without appropriate control groups. No randomized trials were found in the trauma population. Studies also included different transfusion triggers and transfusion protocols, limiting direct comparisons when evaluating prediction for transfusion. Reproducible technical standards for the performance of ROTEM® were lacking in the included studies. Inconsistent reporting data precluded calculation of summary diagnostic test-performance measures and exploration of threshold effects. Different cut-off values and different parameters were used, what makes standardization and interpretation difficult. A major problem faced by diagnostic studies of ACoTS is the ambiguous nature of the gold standard, given that SCTs may not provide an adequate description of all associated abnormalities or may be inferior to ROTEM® parameters. Important treatment differences between many included studies and contemporary practice include substitution of FFP for clotting factors concentrate such as PCC, FC, and cryoprecipitate, what contributed to the clinical heterogeneity across the studies. Although this review found intense heterogeneity, clinically useful and seemingly valid conclusions were reported, and will be useful in designing future studies and future clinical practice guidelines. Our findings add to the current literature importantly, as we were able to summarize and critically appraise the evidence on the threshold values of ROTEM® parameters use in trauma and demonstrate that the accuracy of the current parameters and their cut-off values need further research to be consolidated.

The information obtained in this review may be useful in designing properly and adequately powered clinical trials to detect differences in laboratory and clinical endpoints, such as bleeding, morbidity, and mortality. Confirmation to whether a resuscitation process guided by ROTEM® parameter thresholds will result in less exposure to allogeneic blood products, as compared to resuscitation guided by SCT or with a blind formula resuscitation is still warranted. Additionally, determination of specific ROTEM® parameter thresholds as independently predictors of the need for massive transfusion and mortality, assisting the trauma team with prognostication soon after arrival to hospital, are needed.

## Conclusion

In summary, this systematic review finds that, consistently across all manuscripts reviewed, abnormal EXTEM and FIBTEM clot amplitude (CA5, CA10, CA20) and MCF are capable of diagnosing ACoTS (compared to SCT tests), predict the need for massive transfusion, and predict mortality. Furthermore, the presence of lysis, diagnosed by abnormal LI30 or ML is also strongly associated with mortality. Thus, based on the current available evidence we reviewed, it could be extrapolated that clinical practice guidelines using ROTEM® parameters thresholds to guide blood component transfusion could be clinically useful. Goal-directed component transfusion approach guided by ROTEM® may reduce the exposure to allogeneic blood products and the complications derived from inappropriate resuscitation. However, due to the use of arbitrary cut-off values, lack of randomized controlled trials, cohort studies with small sample sizes, without comparable controls, and heterogeneous patient populations, no further conclusions can be drawn from the literature to date. Better designed prospective studies comparing ROTEM®-guided transfusion protocols with conventional massive transfusion protocols or transfusion guided by SCTs are warranted to determine optimal parameters and accurate thresholds.

## Additional file

Additional file 1: A systematic review on the rotational thrombelastometry (ROTEM®). (DOCX 25 kb)

## Abbreviations

ACoTS: Acute coagulopathy of trauma and shock
ACT: Activated clotting time
APTEM: Aprotinin-based extrinsically activated test
aPTT: Activated partial thromboplastin time
ATC: Acute trauma coagulopathy
AUC: Area under the curve
CA10: Clot amplitude at 10 min
CA15: Clot amplitude at 15 min
CFT: Clot formation time
CI: Confidence interval
CLI: Clot lysis index
CT: Clotting time
ELT: Euglobin lysis time
EXTEM: Extrinsically-activated test
FC: Fibrinogen concentrate
FFP: Fresh frozen plasma
FIBTEM: Fibrin-based extrinsically activated test
HF: Hyperfibrinolysis
INR: International normalized ratio
INTEM: Intrinsically-activated test
IQR: Interquartile range
ISS: Injury severity score
LI30: Lysis index at 30 min
MA: Maximal amplitude
MCF: Maximal clot firmness
ML: Maximum lysis
MT: Massive transfusion
NOS: Newcastle-Ottawa scale
NPV: Negative predictive value
OR: Odds ratio
PCC: Prothrombin complex concentrate
PLT: Platelet concentrate
POC: Point-of-care
PPV: Positive predictive value
PRISMA: Preferred reporting items for systematic reviews and meta-analyses
PT: Prothrombin time
QUADAS: Quality assessment of diagnostic accuracy studies
RBC: Red blood cells
RCT: Randomized controlled trial
RCT: Randomized controlled trial
RISC: Revised injury severity classification
ROC: Receiver operating curve
ROTEM®: Rotational thromboelastometry
r-TEG®: Rapid thromboelastography
SBP: Systolic blood pressure
SCT: Standard coagulation test
SD: Standard deviation
TAT: Turnaround time
TBI: Traumatic brain injury
TEG®: Thromboelastography
TEM: Thromboelastometry
TG: Thrombin generation
TRISS: Trauma injury severity score
UK: United Kingdom
