# Supplementary material for: A systematic review on the rotational thrombelastometry (ROTEM®) values for the diagnosis of coagulopathy, prediction and guidance of blood transfusion and prediction of mortality in trauma patients
Source: Scand J Trauma Resusc Emerg Med. 2016 Oct 3;24:114. doi: 10.1186/s13049-016-0308-2 (PMC5048662; doi:10.1186/s13049-016-0308-2)
Supplement: Additional file 1: — A systematic review on the rotational thrombelastometry (ROTEM®). (DOCX 25 kb) [file 13049_2016_308_MOESM1_ESM.docx]

**Additional file 1**

A systematic review on the rotational thrombelastometry (ROTEM^®^) values for the diagnosis of coagulopathy, prediction and guidance of blood transfusion and prediction of mortality in trauma patients

Precilla V Veigas, Jeannie Callum, Sandro Rizoli,

Bartolomeu Nascimento, Luis Teodoro da Luz

This supplement provides details of the search strategy for this systematic review. It also provides a list of included and excluded studies.

**1 - Data sources and search strategy**

**Database: Ovid MEDLINE(R) <1946 to March Week 2 2016>**

------------------------------------------------------------------------------------

1 exp Thrombelastography/ (4040)

2 (thromb?elastometr* or thromb?elastogra* or (thromb* adj2 elastometr*) or (thromb* adj2 elastogr*) or ROTEM or TEG

or ROTEG).mp. [mp=title, abstract, original title, name of substance word, subject heading word, keyword heading word,

protocol supplementary concept word, rare disease supplementary concept word, unique identifier] (5234)

3 1 or 2 [ROTEM] (5234)

4 Trauma.mp. [mp=title, abstract, original title, name of substance word, subject heading word, keyword heading

word, protocol supplementary concept word, rare disease supplementary concept word, unique identifier] (185523)

5 exp Trauma Centers/ (7479)

6 4 or 5 [TRAUMA] (185523)

7 3 and 6 (365)

8 limit 7 to yr="2015 -Current" (40)

9 remove duplicates from 8 (35)

10 limit 9 to english language (35)

**Database: Embase Classic+Embase <1947 to 2016 Week 12>**

---------------------------------------------------------------------------------------

1 exp Thrombelastography/ (6716)

2 (thromb?elastometr* or thromb?elastogra* or (thromb* adj2 elastometr*) or (thromb* adj2 elastogr*) or ROTEM or TEG

or ROTEG).mp. [mp=title, abstract, heading word, drug trade name, original title, device manufacturer, drug

manufacturer, device trade name, keyword] (9720)

3 1 or 2 [ROTEM] (9720)

4 Trauma.mp. [mp=title, abstract, heading word, drug trade name, original title, device manufacturer, drug

manufacturer, device trade name, keyword] (270348)

5 exp traumatology/ (9989)

6 exp emergency health service/ (76742)

7 4 or 5 or 6 [TRAUMA] (340246)

8 3 and 7 (852)

9 limit 8 to yr="2015 -Current" (122)

10 remove duplicates from 9 (121)

11 limit 10 to english language (119)

12 exp Thrombelastography/ (6716)

13 (thromb?elastometr* or thromb?elastogra* or (thromb* adj2 elastometr*) or (thromb* adj2 elastogr*) or ROTEM or

TEG or ROTEG).mp. [mp=title, abstract, heading word, drug trade name, original title, device manufacturer, drug

manufacturer, device trade name, keyword] (9720)

14 12 or 13 [ROTEM] (9720)

15 Trauma.mp. [mp=title, abstract, heading word, drug trade name, original title, device manufacturer, drug

manufacturer, device trade name, keyword] (270348)

16 exp traumatology/ (9989)

17 exp emergency health service/ (76742)

18 15 or 16 or 17 [TRAUMA] (340246)

19 14 and 18 (852)

20 limit 19 to yr="2015 -Current" (122)

21 remove duplicates from 20 (121)

22 limit 21 to english language (119)

23 21 not 22 (2)

**Database: EBM Reviews - Cochrane Central Register of Controlled Trials <February 2016>**

---------------------------------------------------------------------------------------------------------

1 exp Thrombelastography/ (181)

2 (thromb?elastometr* or thromb?elastogra* or (thromb* adj2 elastometr*) or (thromb* adj2 elastogr*) or ROTEM or TEG

or ROTEG).mp. [mp=title, original title, abstract, mesh headings, heading words, keyword] (417)

3 1 or 2 [ROTEM] (417)

4 Trauma.mp. [mp=title, original title, abstract, mesh headings, heading words, keyword] (6145)

5 exp Trauma Centers/ (134)

6 4 or 5 [TRAUMA] (6145)

7 3 and 6 (17)

8 limit 7 to yr="2015 -Current" (3)

9 remove duplicates from 8 (3)

10 limit 9 to english language (3)

**Database: EBM Reviews - Cochrane Database of Systematic Reviews <2005 to March 16, 2016>**

----------------------------------------------------------------------------------------------------------------

1 (thromb?elastometr* or thromb?elastogra* or (thromb* adj2 elastometr*) or (thromb* adj2 elastogr*) or ROTEM or TEG

or ROTEG).mp. [mp=title, abstract, full text, keywords, caption text] (14)

2 Trauma.mp. [mp=title, abstract, full text, keywords, caption text] (1473)

3 1 and 2 (7)

4 limit 3 to last year (1)

**2 - Articles included in the review**

1. Rugeri L, Levrat A, David JS et al. Diagnosis of early coagulation abnormalities in trauma patients by rotation thrombelastography. J Thromb Haemost 2007;5(2):289-295.

2. Levrat A, Gros A, Rugeri L et al. Evaluation of rotation thrombelastography for the diagnosis of hyperfibrinolysis in trauma patients. Br J Anaesth 2008;100(6):792-797.

3. Schochl H, Frietsch T, Pavelka M, Jambor C. Hyperfibrinolysis after major trauma: differential diagnosis of lysis patterns and prognostic value of thrombelastometry. J Trauma 2009;67(1):125-131.

4. Doran CM, Woolley T, Midwinter MJ. Feasibility of using rotational thromboelastometry to assess coagulation status of combat casualties in a deployed setting. J Trauma 2010;69 Suppl 1:S40-S48.

5. Leemann H, Lustenberger T, Talving P et al. The role of rotation thromboelastometry in early prediction of massive transfusion. J Trauma 2010;69(6):1403-1408

6. Schochl H, Nienaber U, Hofer G et al. Goal-directed coagulation management of major trauma patients using thromboelastometry (ROTEM)-guided administration of fibrinogen concentrate and prothrombin complex concentrate. Crit Care 2010;14(2):R55.

7. Tauber H, Innerhofer P, Breitkopf R et al. Prevalence and impact of abnormal ROTEM(R) assays in severe blunt trauma: results of the 'Diagnosis and Treatment of Trauma-Induced Coagulopathy (DIA-TRE-TIC) study'. Br J Anaesth 2011;107(3):378-387.

8. Schochl H, Solomon C, Traintinger S et al. Thromboelastometric (ROTEM) findings in patients suffering from isolated severe traumatic brain injury. J Neurotrauma 2011;28(10):2033-2041.

9. Schochl H, Cotton B, Inaba K et al. FIBTEM provides early prediction of massive transfusion in trauma. Crit Care 2011;15(6):R265.

10. Davenport R, Manson J, De'Ath H et al. Functional definition and characterization of acute traumatic coagulopathy. Crit Care Med 2011;39(12):2652-2658.

11. Rourke C, Curry N, Khan S et al. Fibrinogen levels during trauma hemorrhage, response to replacement therapy, and association with patient outcomes. J Thromb Haemost 2012;10(7):1342-1351.

12. Woolley T, Midwinter M, Spencer P et al. Utility of interim ROTEM((R)) values of clot strength, A5 and A10, in predicting final assessment of coagulation status in severely injured battle patients. Injury 2013;44(5):593-599.

13. Hagemo JS, Christiaans SC, Stanworth SJ et al. Detection of acute traumatic coagulopathy and massive transfusion requirements by means of rotational thromboelastometry: an international prospective validation study. Critical Care (2015) 19:97

**3 - Studies excluded after full text evaluation**

**3.1. Review articles (8)**

1. Schochl H, Maegele M, Solomon C, Gorlinger K, Voelckel W. Early and individualized goal-directed therapy for trauma-induced coagulopathy. Scand J Trauma Resusc Emerg Med 2012;20:15.

2. Tarmey NT, Woolley T, Jansen JO et al. Evolution of coagulopathy monitoring in military damage-control resuscitation. J Trauma Acute Care Surg 2012;73(6 Suppl 5):S417-S422.

3. Blackbourne LH, Baer DG, Eastridge BJ et al. Military medical revolution: deployed hospital and en route care. J Trauma Acute Care Surg 2012;73(6 Suppl 5):S378-S387.

4. Theusinger OM, Madjdpour C, Spahn DR. Resuscitation and transfusion management in trauma patients: emerging concepts. Curr Opin Crit Care 2012;18(6):661-670.

5. Luddington RJ. Thrombelastography/thromboelastometry. Clin Lab Haematol 2005;27(2):81-90.

6. Johansson PI, Stissing T, Bochsen L, Ostrowski SR. Thrombelastography and tromboelastometry in assessing coagulopathy in trauma. Scand J Trauma Resusc Emerg Med 2009;17:45.

7. Theusinger OM, Levy JH. Point of care devices for assessing bleeding and coagulation in the trauma patient. Anesthesiol Clin 2013;31(1):55-65.

8. Lier H, Vorweg M, Hanke A, Gorlinger K. Thromboelastometry guided therapy of severe bleeding. Essener Runde algorithm. Hamostaseologie 2013;33(1):51-61.

**3.2. Letter (1)**

1. Grassetto A, De NM, Ganzerla B et al. ROTEM(R)-guided coagulation factor concentrate therapy in trauma: 2-year experience in Venice, Italy. Crit Care 2012;16(3):428.

**3.3. Editorial (1)**

1. Spahn DR, Ganter MT. Towards early individual goal-directed coagulation management in trauma patients. Br J Anaesth 2010;105(2):103-105.

**3.4. Case reports (4)**

1. Brenni M, Worn M, Bruesch M, Spahn DR, Ganter MT. Successful rotational thromboelastometry-guided treatment of traumatic haemorrhage, hyperfibrinolysis and coagulopathy. Acta Anaesthesiol Scand 2010;54(1):111-117.
2. Schochl H, Forster L, Woidke R, Solomon C, Voelckel W. Use of rotation thromboelastometry (ROTEM) to achieve successful treatment of polytrauma with fibrinogen concentrate and prothrombin complex concentrate. Anaesthesia 2010;65(2):199-203.
3. Ziegler B, Schimke C, Marchet P, Stogermuller B, Schochl H, Solomon C. Severe Pediatric Blunt Trauma--Successful ROTEM-Guided Hemostatic Therapy with Fibrinogen Concentrate and No Administration of Fresh Frozen Plasma or Platelets. Clin Appl Thromb Hemost 2012.
4. Grassetto A, Saggioro D, Caputo P et al. Rotational thromboelastometry analysis and management of life-threatening haemorrhage in isolated craniofacial injury. Blood Coagul Fibrinolysis 2012;23(6):551-555.

**3.5. Did not meet the eligibility criteria, mixed population, systematic review, comparison of device, comparison of therapy, etc (14)**

1. Theusinger OM, Wanner GA, Emmert MY et al. Hyperfibrinolysis diagnosed by rotational thromboelastometry (ROTEM) is associated with higher mortality in patients with severe trauma. Anesth Analg 2011;113(5):1003-1012.

2. Theusinger OM, Nurnberg J, Asmis LM, Seifert B, Spahn DR. Rotation thromboelastometry (ROTEM) stability and reproducibility over time. Eur J Cardiothorac Surg 2010;37(3):677-683.

3. Tanaka KA, Bolliger D, Vadlamudi R, Nimmo A. Rotational thromboelastometry (ROTEM)-based coagulation management in cardiac surgery and major trauma. J Cardiothorac Vasc Anesth 2012;26(6):1083-1093.

4. Jansen JO, Luke D, Davies E, Spencer P, Kirkman E, Midwinter MJ. Temporal changes in ROTEM(R)-measured coagulability of citrated blood samples from coagulopathic trauma patients. Injury 2013;44(1):36-39.

5. Kutcher ME, Cripps MW, McCreery RC et al. Criteria for empiric treatment of hyperfibrinolysis after trauma. J Trauma Acute Care Surg 2012;73(1):87-93.

6. Reed MJ, Nimmo AF, McGee D et al. Rotational thrombolelastometry produces potentially clinical useful results within 10 min in bleeding emergency department patients: the DEUCE study. Eur J Emerg Med 2013;20(3):160-166.

7. Kashuk JL, Moore EE, Wohlauer M et al. Initial experiences with point-of-care rapid thrombelastography for management of life-threatening postinjury coagulopathy. Transfusion 2012;52(1):23-33.

8. Enriquez LJ, Shore-Lesserson L. Point-of-care coagulation testing and transfusion algorithms. Br J Anaesth 2009;103 Suppl 1:i14-i22.

9. Bartal C, Yitzhak A. The role of thromboelastometry and recombinant factor VIIa in trauma. Curr Opin Anaesthesiol 2009;22(2):281-288.

10. Hoyt DB, Dutton RP, Hauser CJ et al. Management of coagulopathy in the patients with multiple injuries: results from an international survey of clinical practice. J Trauma 2008;65(4):755-764.

11. Solbeck S, Windelov NA, Baek NH, Nielsen JD, Ostrowski SR, Johansson PI. In-vitro comparison of free oscillation rheometry (ReoRox) and rotational thromboelastometry (ROTEM) in trauma patients upon hospital admission. Blood Coagul Fibrinolysis 2012;23(8):688-692.

12. Sankarankutty A, Nascimento B, Teodoro da LL, Rizoli S. TEG(R) and ROTEM(R) in trauma: similar test but different results? World J Emerg Surg 2012;7 Suppl 1:S3.

13. Schochl H, Nienaber U, Maegele M et al. Transfusion in trauma: thromboelastometry guided coagulation factor concentrate-based therapy versus standard fresh frozen plasma-based therapy. Crit Care 2011;15(2):R83.

14. Hunt H, Stanworth S, Curry N, Woolley T et al. Thromboelastography (TEG) and rotational

thromboelastometry (ROTEM) for trauma induced coagulopathy. Cochrane Database of

Systematic Reviews 2015, Issue 2. Art. No.: CD010438
